# Psychological impact of workplace violence towards nurses in the emergency department

**DOI:** 10.1192/j.eurpsy.2023.1838

**Published:** 2023-07-19

**Authors:** A. Koubaa, N. Khouja, S. Ismail, E. Bechrifa, E. Baraketi, A. Ben Jemaa, J. Hsinet, A. Benzarti

**Affiliations:** Service de la Médecine du travail et des Maladies professionnelles, Centre hospitalo-universitaire La Rabta, Tunis, Tunis, Tunisia

## Abstract

**Introduction:**

Growing attention has been drawn to workplace violence in healthcare settings and toward healthcare workers. Emergency departments have always been vulnerable to workplace violence since its is usually associated to working with people in extreme distress. Healthcare workers facing numbers of situation with either verbal or physical abuse may face an impact on their mental health.

**Objectives:**

Describe the psychological impact of workplace violence towards nurses in the emergency department

**Methods:**

This is a questionnaire-based cross-sectional study including nurses working in the emergency department of different hospitals of Tunis. The respondents’ data were collected from March 2022 to September 2022.

**Results:**

We included 164 nurses. They were mainly women (Sex ratio of 0.6) with a median of age was 30.7±7.6 years. Nineteen nurses had history of one or more mental disorders such as anxiety disorder (12 cases) and depressive disorder (11 cases). All nurses were victim of workplace violence, while most (104 nurses) were witness to at least one act of violence. Study population had different degrees of concern about safety in workplace, 29.8% had little to no concern while 29,9% were mildly concerned and 40.2% were severely concerned for which they felt their security compromised (14.6% often and 12.2% always). Violence in workplace had consequences on the quality of their service for 26.8% of nurses. Many (49.4%) felt prejudice on their mental health contrasting with little resort (14%) to mental health providers. Number of nurses presented nightmares (44 cases), avoidance (56 cases) or flashbacks (48 cases) about workplace violence. Psychosomatic symptoms were described by most of the study population (58.5%) such as throbbing (46 cases), nervousness (56 cases), aches (23 cases), fatigue (29 cases) or non-specific digestive signs (17 cases).

**Conclusions:**

Workplace violence in emergency departments, first interface of the care chain, is a real problem with serious consequences. Healthcare workers are forced to provide care in situations that compromise their health and safety. Implementing a national action plan is essential in order to address this dangerous phenomenon.

**Disclosure of Interest:**

None Declared

